# 4-Chloro­anilinium 3-carb­oxy­prop-2-enoate

**DOI:** 10.1107/S1600536812008458

**Published:** 2012-03-03

**Authors:** R. Anitha, S. Athimoolam, S. Asath Bahadur, M. Gunasekaran

**Affiliations:** a Department of Physics, Anna University of Technology Tirunelveli, Tirunelveli 627 007, India; b Department of Physics, University College of Engineering Nagercoil, Anna University of Technology Tirunelveli, Nagercoil 629 004, India; c Department of Physics, Kalasalingam University, Anand Nagar, Krishnan Koil 626 126, India

## Abstract

In the title compound, C_6_H_7_ClN^+^·C_4_H_3_O_4_^−^, the cations and anions lie on mirror planes and hence only half of the mol­ecules are present in the asymmeric unit. The 4-chloro­anilinium cation and hydrogen maleate anion in the asymmetric unit are each planar and are oriented at an angle of 15.6 (1)° to one another and perpendicular to the *b* axis. A characterestic intra­molecular O—H⋯O hydrogen bond, forming an S(7) motif, is observed in the maleate anion. In the crystal, the cations and anions are linked by N—H⋯O hydrogen bonds, forming layers in the *ab* plane. The aromatic rings of the cations are sandwiched between hydrogen-bonded chains and rings formed through the amine group of the cation and maleate anions, leading to alternate hydro­phobic (*z* = 0 or 1) and hydro­philic layers (*z* = 1/2) along the *c* axis.

## Related literature

For related structures, see: Anitha *et al.* (2011 ▶); Balamurugan *et al.* (2010 ▶); Ploug-Sørenson & Andersen (1985 ▶); Rahmouni *et al.* (2010 ▶); Smith *et al.* (2005 ▶, 2007 ▶, 2009 ▶). For the importance of 4-chloro­aniline, see: Ashford (2011 ▶); Amoa (2007 ▶). For hydrogen-bond motifs, see: Bernstein *et al.* (1995 ▶).
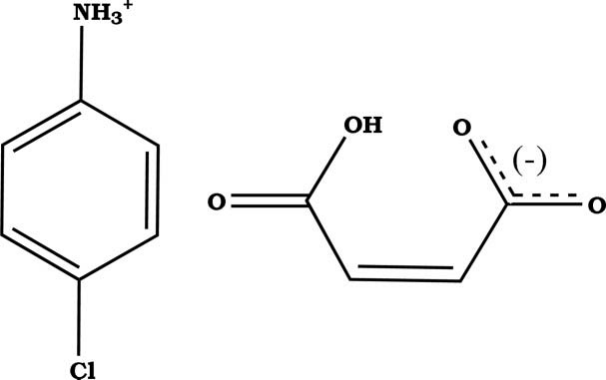


## Experimental

### 

#### Crystal data

C_6_H_7_ClN^+^·C_4_H_3_O_4_^−^
*M* *_r_* = 243.64
Monoclinic, 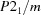

*a* = 3.8932 (3) Å
*b* = 9.1841 (6) Å
*c* = 14.8394 (9) Å
β = 93.664 (12)°
*V* = 529.51 (6) Å^3^
*Z* = 2
Mo *K*α radiation
μ = 0.36 mm^−1^
*T* = 293 K
0.21 × 0.18 × 0.15 mm

#### Data collection

Bruker SMART APEX CCD area-detector diffractometer
5030 measured reflections
998 independent reflections
921 reflections with *I* > 2σ(*I*)
*R*_int_ = 0.026

#### Refinement

*R*[*F* ^2^ > 2σ(*F* ^2^)] = 0.037
*wR*(*F* ^2^) = 0.106
*S* = 1.06
998 reflections
89 parameters
H atoms treated by a mixture of independent and constrained refinement
Δρ_max_ = 0.27 e Å^−3^
Δρ_min_ = −0.18 e Å^−3^

### 

Data collection: *SMART* (Bruker, 2001 ▶); cell refinement: *SAINT* (Bruker, 2001 ▶); data reduction: *SAINT*; program(s) used to solve structure: *SHELXTL/PC* (Sheldrick, 2008 ▶); program(s) used to refine structure: *SHELXTL/PC*; molecular graphics: *PLATON* (Spek, 2009 ▶); software used to prepare material for publication: *SHELXTL/PC*.

## Supplementary Material

Crystal structure: contains datablock(s) global, I. DOI: 10.1107/S1600536812008458/sj5203sup1.cif

Structure factors: contains datablock(s) I. DOI: 10.1107/S1600536812008458/sj5203Isup2.hkl

Supplementary material file. DOI: 10.1107/S1600536812008458/sj5203Isup3.cml

Additional supplementary materials:  crystallographic information; 3D view; checkCIF report

## Figures and Tables

**Table 1:** Hydrogen-bond geometry (Å, °)

*D*—H⋯*A*	*D*—H	H⋯*A*	*D*⋯*A*	*D*—H⋯*A*
N1—H1*N*⋯O2	0.94 (2)	1.87 (2)	2.764 (2)	158 (2)
N1—H2*N*⋯O2^i^	0.82 (4)	2.34 (3)	2.928 (2)	129 (1)
N1—H2*N*⋯O2^ii^	0.82 (4)	2.34 (3)	2.928 (2)	129 (1)
O1—H1*O*⋯O1^iii^	1.21 (1)	1.21 (1)	2.399 (2)	167 (1)

Symmetry codes: (i) 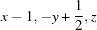; (ii) 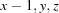; (iii) 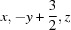.

**Fig. 1.**
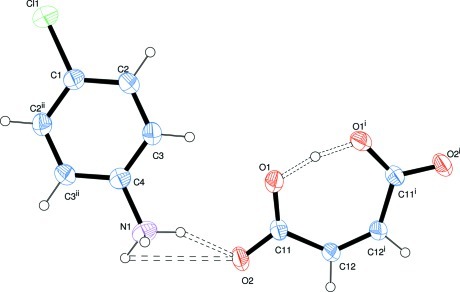
The structure of the title compound (I) with the numbering scheme for the atoms and 50% probability displacement ellipsoids. H bonds are drawn as dashed lines.

**Fig. 2.**
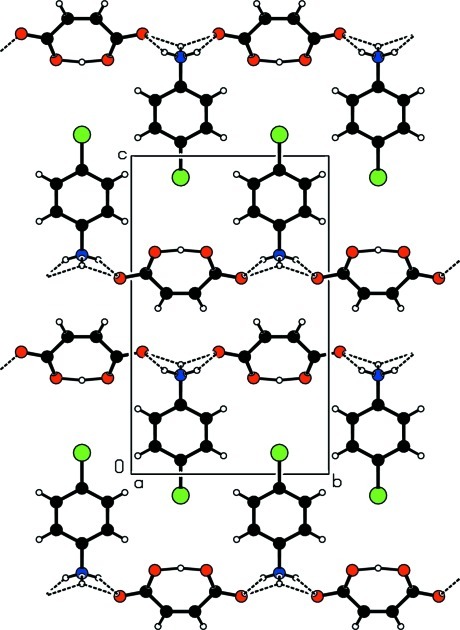
Packing diagram of the molecules viewed down the a-axis. H bonds are drawn as dashed lines.

## supplementary crystallographic information

### Comment

*p*-Chloroaniline is used as an intermediate in the production of several urea herbicides and insecticides (*e.g.*, monuron, diflubenzuron), azo dyes, pigments, pharmaceutical and cosmetic products. It is a precursor to the widely used antimicrobial and bacteriocide chlorhexidine and is used in the manufacture of pesticides, including pyraclostrobin, anilofos, monolinuron and chlorphthalim (Ashford, 2011). Maleic acid can be converted into maleic anhydride by dehydration, to malic acid by hydration, and to succinic acid by hydrogenation (Amoa, 2007). The maleate ion is the ionized form of maleic acid (a monoanion in the present structure). It is useful in biochemistry as an inhibitor of transminase reactions. The maleate ion is used with pheniramine as an antihistamine drug in day-to-day use to treat allergic conditions such as hay fever or urticaria. Also we continuously seek to identify hydrogen bond enriched assemblies by means of a single efficient organic hydrogen bonding synthon. Substituted anilines are good candidates for this type of supramolecular synthon. In a continuation of our previous report on nitro substituted aniline (Anitha *et al.*, 2011), the title compound is presented here derived from a chloro substituted aniline with maleic acid.

As the molecules lie on adjacent mirror planes, the asymmetric unit of the title compound, (I), contains half of a 4-chloroanilinium cation and half of a hydrogen maleate anion (Fig. 1). The bond distances and angles of the cation are comparable with the related 4-chloroanilinium structures (Balamurugan *et al.*, 2010; Ploug-Sørenson & Andersen, 1985; Rahmouni *et al.*, 2010; Smith *et al.*, 2005, 2007, 2009). The planes of the cation and the hydrogen maleate anion are oriented at an angle of 15.6 (1)° to each other. Cations and anions are oriented perpendicular to the *b* axis (mirror plane) of the unit cell. A characterestic intramolecular O—H···O hydrogen bond, forming an S(7) motif, is observed in the maleate anion (Bernstein *et al.*, 1995).

The crystal packing is stabilized through a two dimensional hydrogen bonding network which connects cations and anions through intermolecular N—H···O hydrogen bonds on the *ab*-plane. Cations are linked through anions making a chain C_2_^2^(9) motif extending parallel to the *b* axis of the unit cell through an N1—H1N···O2 hydrogen bond. This leads to molecular aggregations of cations and anions perpendicular to the *ac*-plane of the unit cell. These cationic and anionic molecular aggregations make an angle of 15.7 (1)° to each other. These two-dimensional molecular aggregations are further connected through another two hydrogen bonds, namely N1—H2N···O2^(i)^ and N1—H2N···O2^(ii)^(For symmetry codes: see Table 1), leading to unusal ring *R*_3_^4^(6) motifs which are arranged in tandem along *a* axis of the unit cell. The aromatic rings of the cations are sandwiched between hydrogen bonded chains and rings formed through the amine group of the cations and maleate anions leading to alternate hydrophobic (*z* = 0 or 1) and hydrophilic layers (*z* = 1/2) along *c* axis of the unit cell (Fig. 2). Notably, the electronegative chlorine atom does not participate as an acceptor in any hydrogen bonding interaction.

### Experimental

The title compound was crystallized from an aqueous mixture containing 4-chloroaniline and maleic acid in the stoichiometric ratio of 1:1 at room temperature by the slow evaporation technique.

### Refinement

All the H atoms except the atoms involved in hydrogen bonds were positioned geometrically and refined using a riding model, with C—H = 0.93 Å and *U*_iso_(H) = 1.2 *U*_eq_ (parent atom). H atoms bound to N and O were located in a difference Fourier map and refined isotropically.

### Crystal data

**Table:**

C_6_H_7_ClN^+^·C_4_H_3_O_4_^−^	*F*(000) = 252
*M**_r_* = 243.64	*D*_x_ = 1.528 Mg m^−^^3^*D*_m_ = 1.53 (1) Mg m^−^^3^*D*_m_ measured by Flotation technique using a liquid-mixture of carbon tetrachloride and bromoform
Monoclinic, *P*2_1_/*m*	Mo *K*α radiation, λ = 0.71073 Å
Hall symbol: -P 2yb	Cell parameters from 2243 reflections
*a* = 3.8932 (3) Å	θ = 2.4–24.7°
*b* = 9.1841 (6) Å	µ = 0.36 mm^−^^1^
*c* = 14.8394 (9) Å	*T* = 293 K
β = 93.664 (12)°	Block, colourless
*V* = 529.51 (6) Å^3^	0.21 × 0.18 × 0.15 mm
*Z* = 2	

### Data collection

**Table:**

Bruker SMART APEX CCD area-detector diffractometer	921 reflections with *I* > 2σ(*I*)
Radiation source: fine-focus sealed tube	*R*_int_ = 0.026
Graphite monochromator	θ_max_ = 25.0°, θ_min_ = 2.6°
ω scans	*h* = −4→4
5030 measured reflections	*k* = −10→10
998 independent reflections	*l* = −17→17

### Refinement

**Table:**

Refinement on *F*^2^	Primary atom site location: structure-invariant direct methods
Least-squares matrix: full	Secondary atom site location: difference Fourier map
*R*[*F*^2^ > 2σ(*F*^2^)] = 0.037	Hydrogen site location: inferred from neighbouring sites
*wR*(*F*^2^) = 0.106	H atoms treated by a mixture of independent and constrained refinement
*S* = 1.06	*w* = 1/[σ^2^(*F*_o_^2^) + (0.0648*P*)^2^ + 0.1135*P*] where *P* = (*F*_o_^2^ + 2*F*_c_^2^)/3
998 reflections	(Δ/σ)_max_ < 0.001
89 parameters	Δρ_max_ = 0.27 e Å^−^^3^
0 restraints	Δρ_min_ = −0.18 e Å^−^^3^

### Special details

**Table:**

Geometry. All e.s.d.'s (except the e.s.d. in the dihedral angle between two l.s. planes) are estimated using the full covariance matrix. The cell e.s.d.'s are taken into account individually in the estimation of e.s.d.'s in distances, angles and torsion angles; correlations between e.s.d.'s in cell parameters are only used when they are defined by crystal symmetry. An approximate (isotropic) treatment of cell e.s.d.'s is used for estimating e.s.d.'s involving l.s. planes.
Refinement. Refinement of *F*^2^ against ALL reflections. The weighted *R*-factor *wR* and goodness of fit *S* are based on *F*^2^, conventional *R*-factors *R* are based on *F*, with *F* set to zero for negative *F*^2^. The threshold expression of *F*^2^ > σ(*F*^2^) is used only for calculating *R*-factors(gt) *etc*. and is not relevant to the choice of reflections for refinement. *R*-factors based on *F*^2^ are statistically about twice as large as those based on *F*, and *R*- factors based on ALL data will be even larger.

### Fractional atomic coordinates and isotropic or equivalent isotropic displacement parameters (Å^2^)

**Table:**

	*x*	*y*	*z*	*U*_iso_*/*U*_eq_	
N1	0.1795 (5)	0.2500	0.31121 (14)	0.0544 (5)	
C1	−0.2237 (6)	0.2500	0.04426 (15)	0.0483 (5)	
C2	−0.1599 (4)	0.37969 (18)	0.08748 (12)	0.0548 (4)	
H2	−0.2080	0.4672	0.0577	0.066*	
C3	−0.0243 (4)	0.37952 (18)	0.17517 (11)	0.0523 (4)	
H3	0.0214	0.4668	0.2053	0.063*	
C4	0.0427 (5)	0.2500	0.21759 (14)	0.0443 (5)	
Cl1	−0.38471 (18)	0.2500	−0.06702 (4)	0.0710 (3)	
H1N	0.314 (6)	0.332 (3)	0.3260 (16)	0.094 (8)*	
H2N	0.020 (11)	0.2500	0.344 (3)	0.108 (14)*	
C11	0.6247 (4)	0.57400 (16)	0.36908 (10)	0.0442 (4)	
C12	0.8034 (4)	0.67786 (17)	0.43181 (10)	0.0443 (4)	
H12	0.9374	0.6353	0.4788	0.053*	
O1	0.4349 (3)	0.61939 (12)	0.30237 (9)	0.0630 (4)	
O2	0.6665 (3)	0.44284 (12)	0.38410 (8)	0.0570 (4)	
H1O	0.413 (11)	0.7500	0.295 (3)	0.129 (15)*	

### Atomic displacement parameters (Å^2^)

**Table:**

	*U*^11^	*U*^22^	*U*^33^	*U*^12^	*U*^13^	*U*^23^
N1	0.0412 (10)	0.0708 (15)	0.0505 (11)	0.000	−0.0032 (8)	0.000
C1	0.0444 (11)	0.0501 (13)	0.0498 (12)	0.000	−0.0020 (9)	0.000
C2	0.0614 (10)	0.0408 (10)	0.0610 (10)	−0.0006 (7)	−0.0045 (8)	0.0064 (7)
C3	0.0575 (9)	0.0409 (9)	0.0579 (9)	−0.0049 (7)	−0.0016 (7)	−0.0041 (7)
C4	0.0330 (9)	0.0508 (12)	0.0490 (11)	0.000	0.0017 (8)	0.000
Cl1	0.0805 (5)	0.0748 (5)	0.0552 (4)	0.000	−0.0161 (3)	0.000
C11	0.0455 (8)	0.0350 (8)	0.0520 (9)	0.0001 (6)	0.0026 (6)	−0.0033 (6)
C12	0.0484 (8)	0.0362 (8)	0.0472 (8)	0.0023 (6)	−0.0058 (6)	0.0014 (6)
O1	0.0742 (8)	0.0419 (7)	0.0691 (8)	−0.0007 (6)	−0.0256 (6)	−0.0081 (5)
O2	0.0683 (7)	0.0308 (6)	0.0711 (8)	0.0001 (5)	−0.0017 (6)	−0.0044 (5)

### Geometric parameters (Å, º)

**Table:**

N1—C4	1.456 (3)	C3—H3	0.9300
N1—H1N	0.94 (2)	C4—C3^i^	1.3632 (19)
N1—H2N	0.82 (4)	C11—O2	1.2339 (19)
C1—C2	1.368 (2)	C11—O1	1.2674 (18)
C1—C2^i^	1.368 (2)	C11—C12	1.476 (2)
C1—Cl1	1.728 (2)	C12—C12^ii^	1.325 (3)
C2—C3	1.373 (2)	C12—H12	0.9300
C2—H2	0.9300	O1—H1O	1.207 (5)
C3—C4	1.3632 (19)		
			
C4—N1—H1N	112.8 (15)	C2—C3—H3	120.3
C4—N1—H2N	109 (3)	C3^i^—C4—C3	121.5 (2)
H1N—N1—H2N	107 (2)	C3^i^—C4—N1	119.22 (10)
C2—C1—C2^i^	121.1 (2)	C3—C4—N1	119.22 (10)
C2—C1—Cl1	119.46 (11)	O2—C11—O1	121.72 (14)
C2^i^—C1—Cl1	119.46 (11)	O2—C11—C12	117.76 (14)
C1—C2—C3	119.39 (15)	O1—C11—C12	120.53 (14)
C1—C2—H2	120.3	C12^ii^—C12—C11	130.27 (8)
C3—C2—H2	120.3	C12^ii^—C12—H12	114.9
C4—C3—C2	119.30 (15)	C11—C12—H12	114.9
C4—C3—H3	120.3	C11—O1—H1O	115 (2)
			
C2^i^—C1—C2—C3	1.1 (4)	C2—C3—C4—N1	−178.68 (18)
Cl1—C1—C2—C3	−178.44 (14)	O2—C11—C12—C12^ii^	−179.82 (9)
C1—C2—C3—C4	−0.3 (3)	O1—C11—C12—C12^ii^	0.04 (18)
C2—C3—C4—C3^i^	−0.5 (3)		

Symmetry codes: (i) *x*, −*y*+1/2, *z*; (ii) *x*, −*y*+3/2, *z*.

### Hydrogen-bond geometry (Å, º)

**Table:**

*D*—H···*A*	*D*—H	H···*A*	*D*···*A*	*D*—H···*A*
N1—H1*N*···O2	0.94 (2)	1.87 (2)	2.764 (2)	158 (2)
N1—H2*N*···O2^iii^	0.82 (4)	2.34 (3)	2.928 (2)	129 (1)
N1—H2*N*···O2^iv^	0.82 (4)	2.34 (3)	2.928 (2)	129 (1)
O1—H1*O*···O1^ii^	1.21 (1)	1.21 (1)	2.399 (2)	167 (1)

Symmetry codes: (ii) *x*, −*y*+3/2, *z*; (iii) *x*−1, −*y*+1/2, *z*; (iv) *x*−1, *y*, *z*.
